# Impact of burosumab on kinetics of intact FGF23 values after the removal of a causative tumor in a patient with tumor-induced osteomalacia

**DOI:** 10.1093/jbmrpl/ziag140

**Published:** 2026-08-20

**Authors:** Koki Irie, Kunihiro Sasaki, Koichiro Furukawa, Ruizhi Jiajue, Natsuho Adachi, Soichiro Kimura, Yoshitomo Hoshino, Naoko Hidaka, Hajime Kato, Maki Takeuchi, Sakae Tanaka, Masaomi Nangaku, Taku Saito, Noriko Makita, Nobuaki Ito

**Affiliations:** Division of Nephrology and Endocrinology, The University of Tokyo Hospital, Tokyo 113-8655, Japan; Osteoporosis Center, The University of Tokyo Hospital, Tokyo 113-8655, Japan; Department of Pediatrics, Hachinohe City Hospital, Aomori 031-8555, Japan; Division of Nephrology and Endocrinology, The University of Tokyo Hospital, Tokyo 113-8655, Japan; Division of Nephrology and Endocrinology, The University of Tokyo Hospital, Tokyo 113-8655, Japan; Department of Pediatrics, The University of Tokyo Hospital, Tokyo 113-8655, Japan; Division of Nephrology and Endocrinology, The University of Tokyo Hospital, Tokyo 113-8655, Japan; Osteoporosis Center, The University of Tokyo Hospital, Tokyo 113-8655, Japan; Division of Nephrology and Endocrinology, The University of Tokyo Hospital, Tokyo 113-8655, Japan; Osteoporosis Center, The University of Tokyo Hospital, Tokyo 113-8655, Japan; Division of Nephrology and Endocrinology, The University of Tokyo Hospital, Tokyo 113-8655, Japan; Osteoporosis Center, The University of Tokyo Hospital, Tokyo 113-8655, Japan; Division of Nephrology and Endocrinology, The University of Tokyo Hospital, Tokyo 113-8655, Japan; Osteoporosis Center, The University of Tokyo Hospital, Tokyo 113-8655, Japan; Division of Nephrology and Endocrinology, The University of Tokyo Hospital, Tokyo 113-8655, Japan; Osteoporosis Center, The University of Tokyo Hospital, Tokyo 113-8655, Japan; Department of Orthopedic Surgery, The University of Tokyo Hospital, Tokyo 113-8655, Japan; Division of Nephrology and Endocrinology, The University of Tokyo Hospital, Tokyo 113-8655, Japan; Osteoporosis Center, The University of Tokyo Hospital, Tokyo 113-8655, Japan; Department of Orthopedic Surgery, The University of Tokyo Hospital, Tokyo 113-8655, Japan; Division of Nephrology and Endocrinology, The University of Tokyo Hospital, Tokyo 113-8655, Japan; Osteoporosis Center, The University of Tokyo Hospital, Tokyo 113-8655, Japan; Osteoporosis Center, The University of Tokyo Hospital, Tokyo 113-8655, Japan; Division of Therapeutic Development for Intractable Bone Diseases, Graduate School of Medicine and Faculty of Medicine, The University of Tokyo, Tokyo 113-8655, Japan

**Keywords:** burosumab, fibroblast growth factor-23 (FGF23), half-life, postoperative period, tumor-induced osteomalacia (TIO)

## Abstract

Tumor-induced osteomalacia (TIO) is a paraneoplastic syndrome caused by tumors secreting fibroblast growth factor 23 (FGF23). Burosumab, an anti-FGF23 antibody, is used for the management of TIO. However, burosumab therapy significantly elevates serum intact FGF23 levels, rendering preoperative systemic venous sampling unreliable. This report aims to evaluate the half-life of measured intact FGF23 in a TIO patient treated with burosumab and to elucidate its effect on the kinetics of intact FGF23 levels. A 13-yr-old female who had been receiving burosumab for TIO underwent wide excision of an FGF23-positive phosphaturic mesenchymal tumor located in the right distal femur. Burosumab was discontinued postoperatively, and intact FGF23 levels were measured sequentially at outpatient follow-ups. The half-life was calculated using a single-phase exponential decay model based on postoperative days and measured intact FGF23 levels. Although hypophosphatemia resolved following tumor removal, measured intact FGF23 levels remained high (44 300 pg/mL on postoperative day 1) but gradually declined and normalized (29.5 pg/mL on postoperative day 271). Based on these measurements, the half-life of measured intact FGF23 was calculated to be 20.2 d. This half-life is similar to that of burosumab itself, likely reflecting the presence of biologically inactive FGF23–burosumab complexes. In conclusion, the half-life of measured intact FGF23 following burosumab discontinuation is considerably longer than that reported in TIO patients not receiving burosumab, indicating that measured intact FGF23 levels should be interpreted with caution after discontinuation of burosumab.

## Graphical Abstract

**Graphical Abstract:**
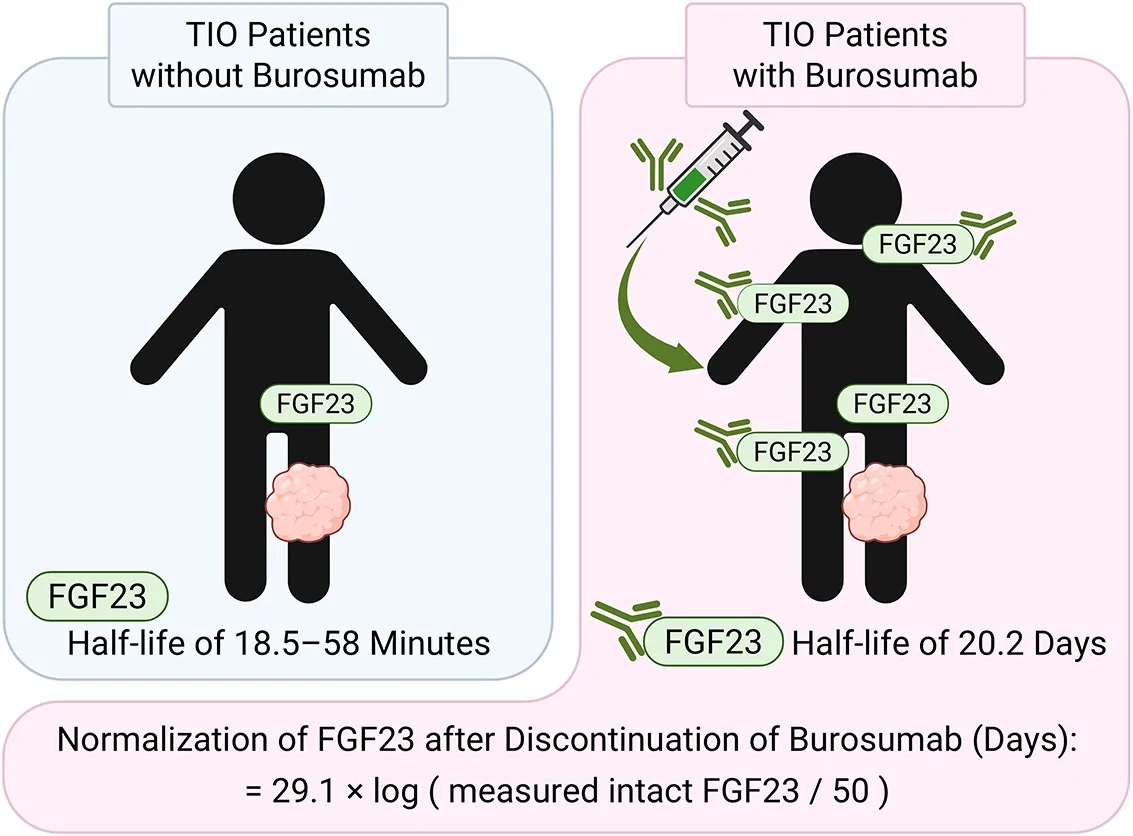

## Introduction

Tumor-induced osteomalacia (TIO) is a rare paraneoplastic syndrome caused by phosphaturic mesenchymal tumors (PMTs) that develop in bone and soft tissues and secrete fibroblast growth factor 23 (FGF23). FGF23 is physiologically produced by the osteocytes, and inhibits renal phosphate reabsorption by binding to the Klotho–FGF receptor 1 (FGFR1) complex and suppressing the expression of sodium–phosphate cotransporters in the renal proximal tubules.^1^ In addition, it regulates vitamin D metabolism by inhibiting CYP27B1 and upregulating CYP24A1 expression, resulting in reduced levels of active 1,25(OH)_2_D_3_.^1^ Overproduction of FGF23 causes chronic hypophosphatemia by decreasing renal phosphate reabsorption, leading to impaired bone mineralization, a condition called osteomalacia. Osteomalacia is characterized by clinical manifestations including pseudofractures, musculoskeletal pain, and fatigue.^1^

In the diagnosis of TIO, osteomalacia is diagnosed based on clinical manifestations and radiographic findings such as those described above. Biochemical evaluation should demonstrate chronic hypophosphatemia with renal phosphate wasting, as assessed by decreased tubular reabsorption of phosphate and tubular maximum reabsorption of phosphate per glomerular filtration rate (TmP/GFR). FGF23-related hypophosphatemia is diagnosed by demonstrating an inappropriately elevated intact FGF23 level in the setting of chronic hypophosphatemia.^1^ According to guidelines published in Japan, FGF23-related hypophosphatemia is diagnosed by demonstrating an inappropriately elevated intact FGF23 level (≥30 pg/mL), as measured using the Determiner CL FGF23 (Canon Medical Diagnostics Corporation) in the setting of chronic hypophosphatemia.^2^ Two types of FGF23 assays are currently available: the intact FGF23 assay and the C-terminal FGF23 (C-FGF23) assay.^1^ The intact FGF23 assay specifically detects the full-length form of FGF23, which is the biologically active form. In contrast, the C-terminal assay detects both intact FGF23 and inactive C-FGF23 fragments. Therefore, the measurement of intact FGF23 is more clinically relevant for assessing the disease activity. To identify the FGF23-producing PMTs, several functional imaging modalities have been used including ^18^F-fluoro-2-deoxy-D-glucose PET/CT (FDG-PET/CT), ^111^In-pentetreotide scintigraphy, ^68^Gallium (^68^Ga)-DOTATOC PET/CT, ^68^Ga-DOTANOC PET/CT, and ^68^Ga-DOTATATE PET/CT, followed by preoperative CT and MRI to characterize the tumor.^1^^,^^3–5^ Moreover, previous studies have demonstrated the usefulness of systemic venous sampling for FGF23.^6–9^ In particular, Kato et al. demonstrated that, among 18 patients with PMTs, venous sampling for FGF23 showed higher sensitivity than FDG-PET/CT or ^111^In-pentetreotide scintigraphy alone.^9^

Complete resection of the causative tumor is the only curative treatment for TIO.^1^ However, localization of the causative tumor is often difficult because most PMTs are benign, slow-growing, and sometimes tiny. Burosumab, a human monoclonal anti-FGF23 antibody, is now an effective treatment option for patients with unresectable, unidentifiable, or recurrent TIO. The therapeutic efficacy of burosumab was initially demonstrated in the treatment of X-linked hypophosphatemia (XLH). Its efficacy has been established in patients with TIO, particularly in cases involving tumors not amenable to surgical resection, tumors that could not be localized, or postoperative recurrence.^1^^,^^10^^,^^11^ However, several issues remain regarding the use of burosumab in patients with TIO. One important issue is that most countries do not yet have burosumab approved for TIO, and in some countries, its use for TIO depends on the type of health insurance coverage. Furthermore, since burosumab is not a curative treatment, patients require long-term administration of this costly drug. Another important issue is the marked elevation of serum intact FGF23 levels observed in patients receiving burosumab. Consequently, venous sampling for FGF23 may no longer be an appropriate diagnostic option after initiation of burosumab therapy.

Previous reports describing patients who underwent surgery for TIO during burosumab treatment showed that measured intact FGF23 or C-FGF23 levels remained significantly elevated for more than 1 mo after surgery.^12^^,^^13^ Nagata et al. reported that measured intact FGF23 levels remained elevated at 4533 pg/mL on postoperative day 64 after successful tumor resection (preoperative: 85 387 pg/mL), with no normalization observed during postoperative follow-up.^12^ Yeh et al. reported that measured C-FGF23 levels (reference value: <180 RU/mL) decreased to 1338 RU/mL at 2 mo and 178 RU/mL at 6 mo after PMT removal.^13^ However, the half-life was not specifically determined in either report.

This study estimates the elimination half-life of “measured” intact FGF23 after discontinuation of burosumab, using serial postoperative measurements in a patient with TIO whose causative tumor was identified during burosumab therapy and subsequently resected. Based on this estimate, the current report further aims to provide clinically useful information for predicting the time required for measured intact FGF23 levels to normalize after discontinuation of burosumab.

## Subject and methods

### Case presentation

The patient was a 13-yr-old female who had experienced bilateral gonalgia and a waddling gait for over 2 yr. The patient had no family history of metabolic bone diseases, and her growth and development history was unremarkable. Initial laboratory data are summarized in Table 1. At diagnosis, laboratory testing showed hypophosphatemia (2.9 mg/dL; reference range, 3.6-5.8 mg/dL for 13-yr-old children), decreased tubular maximum reabsorption of phosphate per glomerular filtration rate (TmP/GFR; 2.3 mg/dL), high normal alkaline phosphatase (400 U/L; 105-483 U/L), and markedly elevated intact FGF23 (194.0 pg/mL; 10-50 pg/mL), indicating FGF23-related hypophosphatemia. Despite the patient’s history of childhood onset, her X-rays revealed no typical physical signs of rickets, and the genetic analyses of *PHEX, FGF23*, and other relevant genes for hereditary FGF23-related hypophosphatemia identified no pathogenic variants. On the other hand, initial imaging studies, including FDG-PET/CT, had failed to detect a tumor associated with TIO.

**Table 1:** Biochemical findings at diagnosis.

	Result	Reference range ^a^
**Serum phosphate (mg/dL)**	2.9	3.6-5.8
**Albumin-corrected calcium (mg/dL)**	9.4	8.7-10.1
**Serum creatinine (mg/dL)**	0.46	0.40-0.70
**Intact FGF23 (pg/mL)**	194	10-50
**Intact PTH (pg/mL)**	76	15-65
**ALP (U/L)**	400	105-483
**Bone-specific ALP (μg/L)**	117	2.9-14.5^*^
**TRACP-5b (mU/dL)**	1173	120-420
**25OHD (ng/mL)**	11.6	>20
**1,25(OH)**_**2**_ **D**_**3**_ **(pg/mL)**	82	20-60
**TRP (%)**	79.8	80-95
**TmP/GFR (mg/dL)**	2.3	2.6-4.4^*^
**Urinary phosphate (mg/dL)**	121.4	-
**Urinary creatinine (mg/dL)**	95.2	-
**Urinary calcium (mg/dL)**	6.0	-

^a^Age- and sex-specific pediatric reference ranges are shown where available; values marked with ^*^indicate adult reference ranges. Abbreviations: ALP, alkaline phosphatase; FGF23, fibroblast growth factor 23; TmP/GFR, tubular maximum reabsorption of phosphate per glomerular filtration rate; TRACP-5b, tartrate-resistant acid phosphatase-5b; TRP, tubular reabsorption of phosphate.

**Figure 1:**
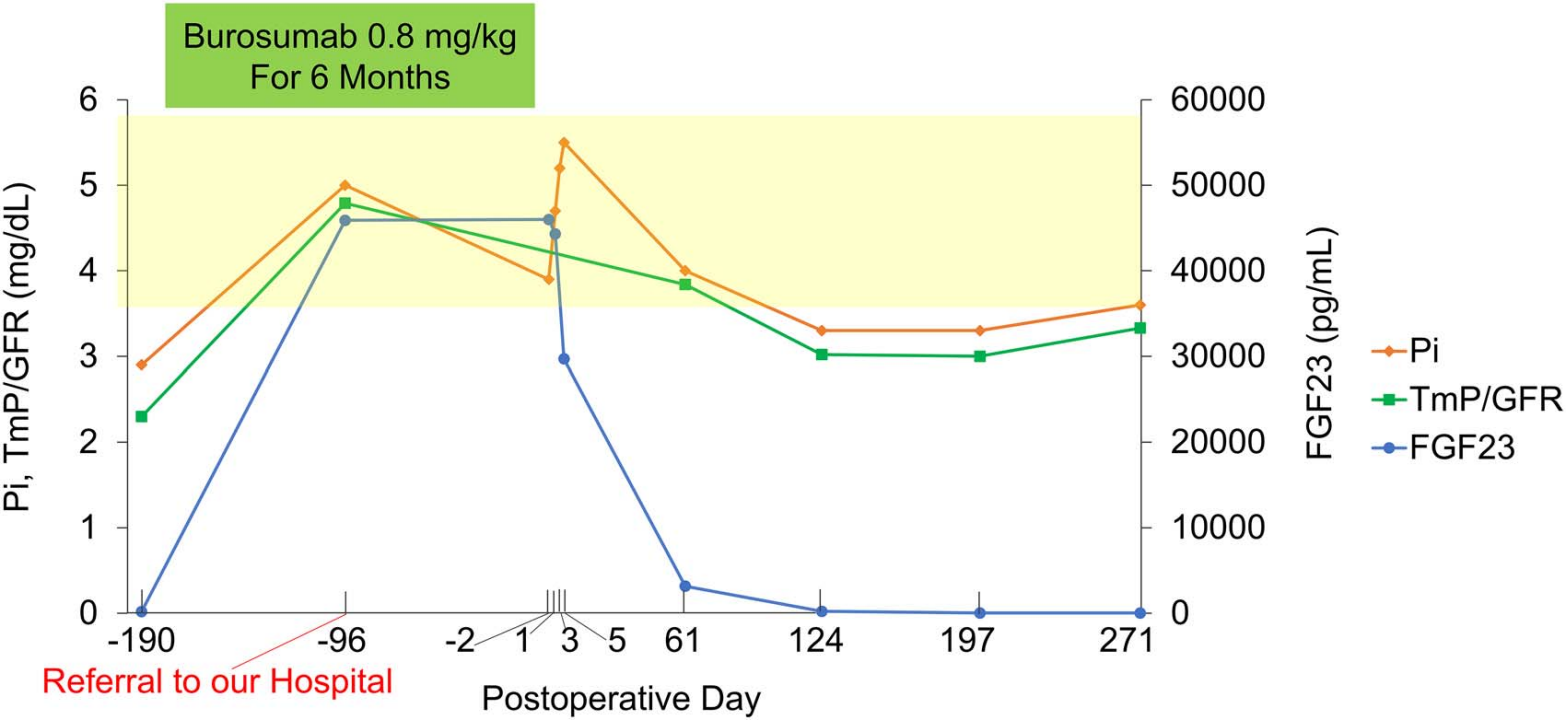
Main biochemical parameters before and after surgical tumor removal. The reference range for serum phosphate (3.8-5.6 mg/dL) is indicated by the shaded area. FGF23, fibroblast growth factor 23; Pi, inorganic phosphate; TmP/GFR, tubular maximum reabsorption of phosphate per glomerular filtration rate.

**Figure 2:**
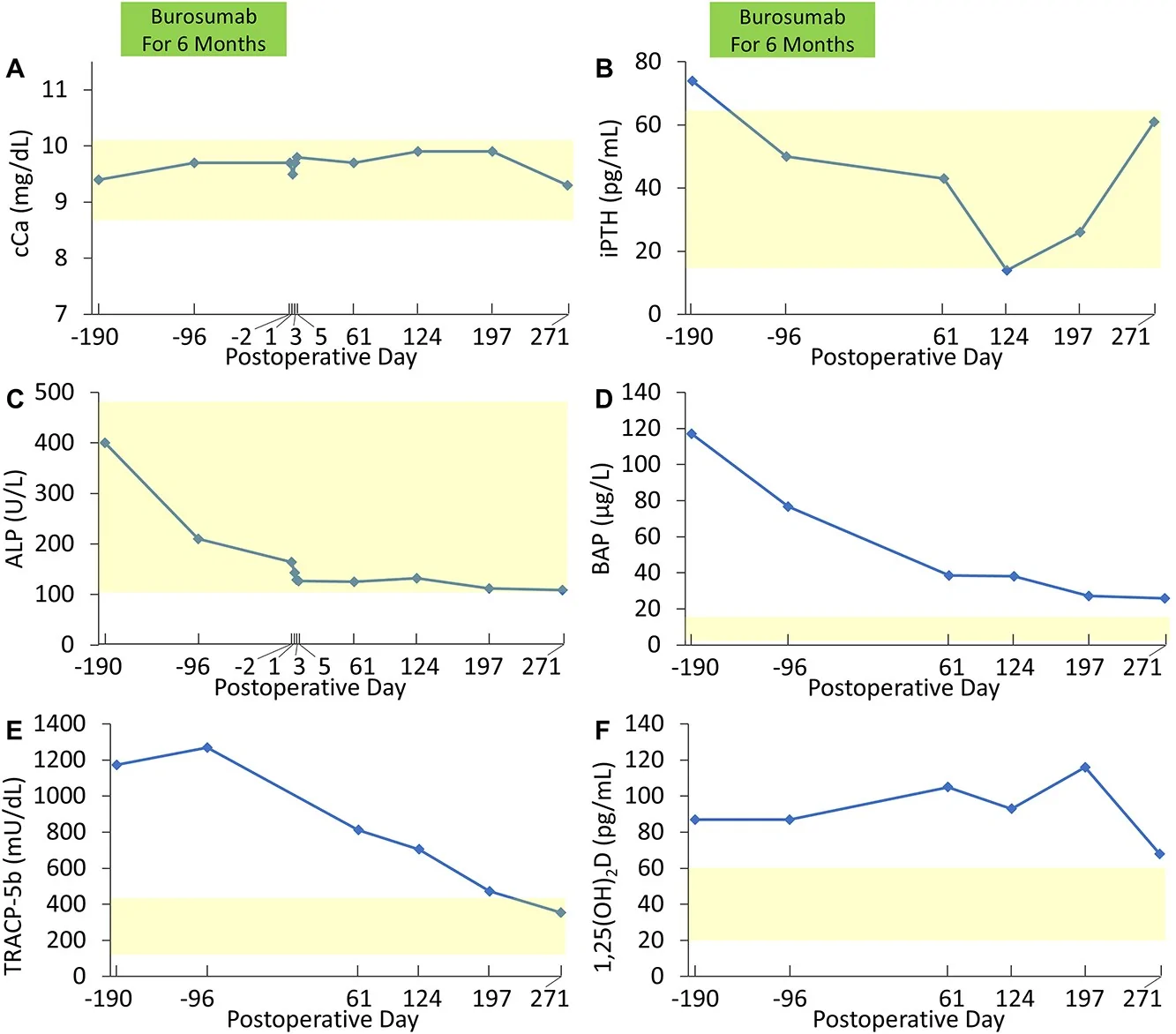
Other biochemical parameters before and after surgical tumor removal: (A) cCa, (B) iPTH, (C) ALP, (D) BAP, (E) TRACP-5b, and (F) 1,25(OH)_2_D_3_. The shaded areas indicate the reference ranges. ALP, alkaline phosphatase; BAP, bone-specific ALP; cCa, albumin-corrected calcium; iPTH, intact PTH; TRACP-5b, tartrate-resistant acid phosphatase 5b.

She was initially treated with alfacalcidol and inorganic phosphate by her attending doctor at the local hospital, followed by a 40 mg (0.8 mg/kg) dose of burosumab every 2 wk as a targeted medical treatment for FGF23-related hypophosphatemia due to either XLH or TIO. Burosumab administration successfully improved her symptoms, normalized serum phosphate levels, and gradually decreased alkaline phosphatase as well as other markers of bone turnover (Figures 1 and 2). To clarify the underlying cause, she was referred to our hospital. Reevaluation of the previous FDG-PET/CT scan revealed asymmetrical uptake in the right distal femur. Contrast-enhanced CT and MRI confirmed a 13-mm tumor located in the lateral aspect of the right femur. Systemic venous sampling for FGF23 was not performed because measured intact FGF23 levels were markedly elevated after burosumab administration. Further details of the diagnostic process have been reported previously.^14^

### Assays

Biochemical tests, including serum phosphate and serum creatinine level measurements, were performed with a LABOSPECT008 (Hitachi). Serum intact FGF23 levels were determined using a chemiluminescent enzyme immunoassay (CLEIA) for FGF23 (Minaris Medical), which exclusively detects full-length FGF23. Postoperative serum samples were collected at outpatient follow-up until measured intact FGF23 levels decreased and normalized to <50 pg/mL.

The relationship between postoperative measured intact FGF23 levels and time after surgery was analyzed using a single-phase exponential decay model:


$$y=a\times{e}^{-b\times x}$$

where *e* is the base of the natural logarithm, *x* represents the postoperative day (POD), and *y* represents the measured intact FGF23 level. The half-life of measured intact FGF23 (*T*) was calculated when $a\times{e}^{-b\times \left(x+T\right)}=0.5\times \left(a\times{e}^{-b\times x}\right)$. In addition, the time required for measured intact FGF23 levels to normalize (upper limit of the reference range: 50 pg/mL) was estimated by solving ${F}_0\times{e}^{-b\times x}=50$, where *F*_0_ represents the measured intact FGF23 level at the reference time point, and *x* represents the number of days until normalization.

## Results

The patient had received burosumab treatment for six months, with the final dose administered 6 d before surgery. After wide excision of the tumor and discontinuation of burosumab, the serum phosphate level normalized to 4.0 mg/dL. However, measured intact FGF23 levels remained high: 46 000 pg/mL 2 d before surgery, 44 300 pg/mL on POD 1, and 29 700 pg/mL on POD 5. Further pathological evaluation confirmed that the tumor was an FGF23-positive PMT, establishing the final diagnosis of TIO.

**Figure 3:**
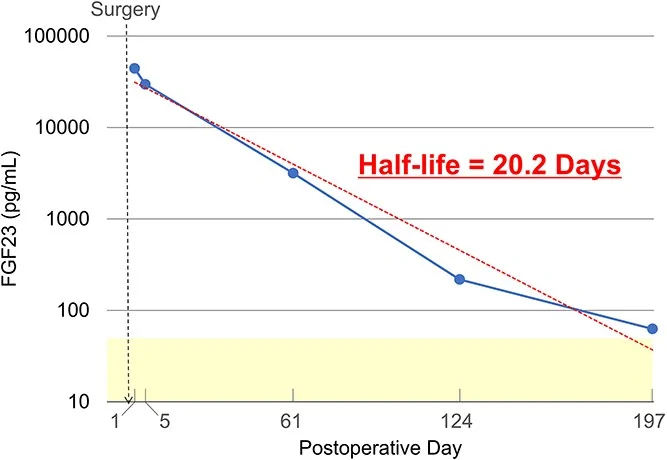
Postoperative changes in measured intact FGF23 and calculation of half-life. The reference range for serum intact FGF23 (10-50 pg/mL) is indicated by the shaded area. The relationship between measured intact FGF23 levels before and after surgery and the time of blood collection represented the following exponential formula: $y=32388\times{e}^{-0.0344\times x}$, where *e* is the base of the natural logarithm, *x* represents the postoperative day, and *y* represents the measured intact FGF23 level. The half-life of measured intact FGF23 (*T*) was calculated when $32388\times{e}^{-0.0344\times \left(x+T\right)}=0.5\times \left(32388\times{e}^{-0.0344\times x}\right)$. FGF23: fibroblast growth factor 23.

After removal of the tumor, blood tests, including serum phosphate and intact FGF23 levels, were performed over time. Serum phosphate levels and other biochemical markers of bone turnover remained stable throughout the postoperative follow-up period, with no evidence of disease recurrence (Figures 1 and 2). During outpatient follow-up, measured intact FGF23 levels gradually declined toward the normal range but remained elevated until nearly 200 d after surgery. On POD 271, the measured intact FGF23 level normalized to 29.5 pg/mL. Using the measurements obtained from POD 1 to POD 197, the final measurement before POD 271, the following exponential approximation line was obtained: $y=32388\times{e}^{-0.0344\times x}$, and the half-life of measured intact FGF23 was calculated to be 20.2 d (Figure 3). Additionally, based on this half-life, a formula was derived to estimate the number of days required for measured intact FGF23 levels to normalize after discontinuation of burosumab (*x*):


\begin{eqnarray*} &x\ \left(\mathrm{days}\ \mathrm{until}\ \mathrm{normalization}\ \mathrm{of}\ \mathrm{FGF}23\right)\nonumber\\&\quad=29.1\times \log \frac{\mathrm{measured}\ \mathrm{intact}\ \mathrm{FGF}23\ \left(\mathrm{pg}/\mathrm{mL}\right)}{50} \nonumber\end{eqnarray*}

where log denotes the natural logarithm.

## Discussion

Typically, complete resection of PMT is the definitive treatment for TIO, and intact FGF23 levels rapidly return to normal after surgery in patients who are not receiving burosumab. Previous studies have reported half-lives of intact FGF23 in the absence of burosumab to be 21.5 min,^15^ 58 min,^16^ and 18.5 min.^17^ However, in patients with burosumab treatment, measured intact FGF23 levels are significantly elevated, and the half-life is markedly prolonged. In fact, a half-life of 20.2 d was derived from the current result (Figure 3). Based on the results without burosumab treatment mentioned above, intact FGF23 levels in this case should have returned from the preoperative levels (approximately 46 000 pg/mL) to normal (less than 50 pg/mL) within 190, 513, or 164 min after surgery, respectively, using the following formula: $50=46000\times{0.5}^{\frac{x}{T}}$, where *T* is the half-life of serum FGF23 (minutes) and *x* indicates the postoperative time (minutes).

Burosumab, a human monoclonal anti-FGF23 antibody, has been demonstrated to significantly increase measured intact FGF23 levels. This increase is hypothesized to result from the formation of FGF23–burosumab complexes, which delay the clearance of FGF23 from the circulation.^18^ In patients with TIO receiving burosumab, serum phosphate and alkaline phosphatase levels can normalize despite markedly elevated measured intact FGF23 levels. Although FGF23–burosumab complexes cannot be directly measured using the current assay, this dissociation is consistent with the hypothesis that free, biologically active intact FGF23 accounts for only a limited fraction of measured intact FGF23. In addition, the calculated half-life of 20.2 d is more consistent with the known terminal half-life of burosumab itself (approximately 19 d)^19^ than with the reported half-life of intact FGF23 in the absence of burosumab (18.5-58 min),^15–17^ supporting the hypothesis that the measured intact FGF23 predominantly reflects the FGF23–burosumab complex. Therefore, when considering the normalization of measured intact FGF23 levels after TIO surgery during burosumab administration, the half-life of the FGF23 complex should be considered.

In this case, serum phosphate levels significantly increased within several days after surgery (Figure 1). In clinical practice, monitoring serum phosphate levels is of primary importance in postoperative patients with TIO.^20^ During burosumab treatment, measured intact FGF23 levels are markedly elevated, likely due to the formation of complexes with burosumab and consequent prolongation of its half-life. As a result, measurement of intact FGF23 has limited clinical utility, and measured intact FGF23 levels should not be considered a reliable indicator of surgical cure in patients previously treated with burosumab. However, when interpreted together with the estimated elimination half-life, measured intact FGF23 levels may still be useful for estimating the time required for these levels to normalize after discontinuation of burosumab. This approach is particularly useful when scheduling systemic venous sampling for FGF23 to reevaluate the localization of the causative tumor in patients with TIO receiving burosumab. A previous report on the postoperative course of TIO patients receiving burosumab indicated that intact FGF23 levels decreased to approximately 1/19 of preoperative levels by 64 d after surgery.^12^ The half-life of 20.2 d calculated in the present case is broadly consistent with the previous observation. Yeh et al. also reported persistent elevation of C-FGF23 levels after surgery in a patient receiving burosumab.^13^ However, in both previous reports, postoperative FGF23 measurements were limited in number. Accordingly, the present serial measurements may provide more detailed information on the postoperative kinetics of measured intact FGF23 after discontinuation of burosumab.

For clinicians, based on the half-life of 20.2 d obtained in this case, a formula was derived to estimate the number of days required for measured intact FGF23 levels to normalize after discontinuation of burosumab; the derivation and specific formula are provided at the end of the Results section. This formula may be useful for determining the timing of venous sampling for FGF23 in situations such as the following: first, when no tumor is identified using imaging diagnostics such as ^68^Ga-DOTATOC PET/CT, and subsequently, burosumab has been started, but reevaluation of tumor localization becomes necessary; and second, when a suspected tumor is identified during burosumab treatment, and further evaluation is required to determine whether it is responsible for FGF23 overproduction. Although this formula provides a practical estimate, further studies are required to address potential interindividual variability because the elimination coefficient was estimated from a single case. While preoperative measured intact FGF23 levels may be influenced by the dose and duration of burosumab treatment, the half-life itself is likely independent of these factors.

In patients receiving burosumab, measured intact FGF23 levels often increase to several tens of thousands of pg/mL. According to the formula derived in the present study, the estimated time required for measured intact FGF23 levels to normalize is approximately 154 d when the level at the reference time point is 10 000 pg/mL and approximately 221 d when it is 100 000 pg/mL. Therefore, the time required for normalization depends on the measured intact FGF23 level at the reference time point and may extend over several months after discontinuation of burosumab.

In conclusion, the half-life of measured intact FGF23 after discontinuation of burosumab was approximately 20 d in a patient with TIO after complete resection of the causative tumor. Therefore, in TIO patients previously treated with burosumab, if systemic venous sampling for FGF23 is to be performed for clinical purposes such as reevaluating tumor localization or confirming tumor-derived FGF23 overproduction, the timing of sampling should be determined in consideration of the prolonged half-life of measured intact FGF23.

## Data Availability

The datasets generated and/or analyzed during the current study are not publicly available but are available from the corresponding author upon reasonable request.
